# Preoperative serum CA 72.4 as prognostic factor of recurrence and death, especially at TNM stage II, for colorectal cancer

**DOI:** 10.1186/1471-2407-13-543

**Published:** 2013-11-12

**Authors:** Daniel Ayude, Francisco Javier Rodríguez-Berrocal, José Ayude, Sonia Blanco-Prieto, Lorena Vázquez-Iglesias, Marta Vázquez-Cedeira, María Páez de la Cadena

**Affiliations:** 1 Department of Biochemistry, Genetics and Immunology, University of Vigo, Vigo, Spain; 2 Department of Biological Production, CZV S.A, 36410, Porriño, Pontevedra, Spain; 3 Department of Computer Sciences, University of Vigo, Vigo, Spain; 4 Institute of Molecular and Cellular Biology of Cancer, CSIC-University of Salamanca, Salamanca, Spain

**Keywords:** Colorectal cancer, Prognosis, Survival, CA 72.4, CEA

## Abstract

**Background:**

Nowadays, evaluation of colorectal cancer prognosis and decision-making for treatment continues to be based primarily on TNM tumour stage. Administration of adjuvant chemotherapy is especially challenging for stage II patients that can have very different disease-related outcomes. Therefore, more reliable prognostic markers need to be developed to improve the selection of stage II patients at high risk for recurrence. Our purpose is to assess the prognostic value of preoperative serum CA 72.4 to improve the risk stratification of CRC patients.

**Methods:**

Preoperative sera collected from 71 unselected patients between January 1994 and February 1997 was assayed for CA 72.4 and CEA levels. Patients were followed-up for at least 30 months or until relapse. Survival curves were estimated by the Kaplan-Meier method and the prognostic value was determined using Log-Rank test and Cox regression analysis.

**Results:**

Preoperative CA 72.4 levels above 7 U/mL correlate with a worse prognosis, with associated recurrence and death percentages exceeding the displayed by CEA. In a multivariate analysis, its combination with CEA proved the most important independent factor predicting survival. Remarkably, at stage II CA 72.4 also discriminates better than CEA those patients that will relapse or die from those with a favourable prognosis; however, CEA has not a negligible effect on survival.

**Conclusions:**

The most outstanding finding of the present work is the correct classification of nearly every patient with bad prognosis (relapse or death) at TNM stage II when CEA and CA 72.4 are used altogether. This could improve the decision-making involved in the treatment of stage II colon cancer. Certainly further large-scale studies must be performed to determine whether CA 72.4 can be effectively used in the clinical setting.

## Background

Colorectal cancer (CRC) is the principal in Europe and the third in United States most commonly diagnosed malignancy in both sexes, and rates second and third origin of cancer-related death in those areas, respectively [1,2].

Long has been investigated to propose novel useful independent prognosticators for CRC [3,4], however, none has been yet integrated into routine practice and prognosis remains an unresolved question in CRC management. Consequently, although some improvement in CRC survival has been recently achieved due to advances in diagnostic and surgical procedures, it continues to be poor [5,6].

At the moment, prognosis for CRC relies mainly on tumour stage [7,8]. Furthermore, the decision of giving adjuvant chemotherapy is based primarily on tumour stage too, except for advanced disease, where some improvement is seen towards more personalized, tumour-specific treatment [9]. TNM (Tumour, Node and Metastasis) stage I disease carries an excellent prognosis, approximately 93% 5-year survival rate [10], and at present there are no convincing data to support adjuvant chemotherapy for patients at this early stage [11]. For stage III colon cancer patients, exhibiting survival rates of 44-83% [10], post-operative chemotherapy is recommended as standard therapy provided its value improving disease free (DFS) and overall survival (OS) [12]. Conversely, patients bearing stage II TNM tumours, with 5-year survival rates ranging from 72 to 85% [10], can experience very different disease-related outcomes and meta-analyses regarding effectiveness of adjuvant therapy in this setting are controversial [12,13]. While some patients experience full recovery after surgical removal of the tumour, others suffer from disease recurrence and metastasis. Thus, the risk exists that patients who would be cured with surgery alone are being subjected to the toxicity of chemotherapy. In this scenario, administration of adjuvant chemotherapy to stage II patients represents the most challenging aspect in the treatment of colon cancer today [14].

We present and discuss new results regarding the value of preoperative serum Carbohydrate Antigen CA 72.4, CA 72.4, in the prognosis of CRC to improve the risk stratification of patients. CA 72.4 has been previously proposed as a serum prognostic tumour marker in gastrointestinal malignancies [15-18].

We have also compared and combined the value of CA 72.4 with the most widely used serum prognostic tumour marker in colorectal cancer, Carcinoembryonic Antigen, CEA, [19-21], and checked whether they represent independent prognostic factors regarding the TNM classification and other patient and tumour features.

Our results suggest that it is worthy to determine preoperative levels of CA 72.4 for an accurate distinction of high-risk patients that should be given chemotherapy, and of low-risk patients who will not have recurrent disease.

## Methods

### Patient and tumour characteristics

Preoperative blood was collected from 137 consecutive unselected patients between January 1994 and February 1997, operated for CRC at “Complejo Hospitalario Universitario de Vigo”, Spain. Whole surgical specimens from tumour and normal mucosa were also obtained from the same patients.

The study was approved by the Ethical Committee of “Complejo Hospitalario Universitario de Vigo” and followed the clinical-ethical practices of the Spanish Government, complied with the Helsinki Declaration, Oviedo Agreement, the Organic Law for Data Protection 15/1999, and Royal Decree 1720/2007. All participants gave informed consent to provide samples and anonymity was warranted using clinical history numbers.

For survival analyses, exclusion criteria included: death within 30 days of surgery, administration of adjuvant therapy either pre- or post-operatively (with the exception of radiotherapy in rectum carcinoma patients), presence of extraganglionar metastases, failure to resect the whole tumour mass, presence of familial adenomatous polyposis coli, inflammatory bowel disease, no adenocarcinoma histology or previous CRC.

Complete prognostic information was obtained on 71 potentially cured patients, TNM stages I-III, that satisfied the inclusion criteria. These patients were followed-up for at least 30 months or until relapse.

The patient group consisted of 38 men and 33 women, with a mean age of 67 (ranging 41–87). The primary tumour location was the colon for 46 patients and the rectum for 25. Regarding tumour grade 4 patients had well differentiated tumours, 61 moderately differentiated tumours and 6 patients presented poorly differentiated tumours. TNM classification was applied to define tumour stage, as follows: 9 patients were classified as having TNM I tumours, 40 patients with TNM II and 22 patients with TNM III.

### TNM classification

All primary colorectal tumours were adenocarcinoma. Surgical specimens were processed for regular pathological and histological examination. Stage of disease was reported according to TNM classification [22].

### Preparation of samples

The drawn blood was allowed to coagulate at room temperature and centrifuged at 2000 g for 15 min. The sera obtained were stored at -85°C until analysis.

### Tumour markers assays

CEA and CA 72.4 were analysed in serum using the commercial immunoassays Enzymun-Test^©^ CEA and Enzymun-Test^©^ CA 72.4 (Boehringer Mannhein, Mannhein). For CA 72.4 we have used a cut-off value of 7 U/mL, and for CEA 10 ng/mL, following the clinical routine at the Hospital and in accordance with other authors [23].

### Statistical methods

A postoperative follow-up of the patients was carried out in order to evaluate the impact of each tumour marker on the disease free survival (D.F.S) and the overall survival (O.S.). Survival curves were estimated by the univariate Kaplan-Meier method. DFS and OS were defined as the time interval from the initial event (curative surgery) to the respective end-points (relapse or death), as well as the time interval from the initial event to the last surveillance date for patients that had not suffered relapse or death. To check the significant differences in the curves among groups the Log-Rank test was applied. Furthermore, univariate and multivariate Cox analysis were performed. All tests were carried out using the Statistical Package for the Social Sciences (SPSS v. 15.0, Chicago, IL). *P* values <0.05 were considered statistically significant.

## Results

A survival study was carried out in 71 curatively tumour resected colorectal cancer patients. After a mean post-operative follow-up period of 44 months, 17 patients presented recurrence (23.9%) and 11 finally died (15.5%). The mean DFS and OS were of 52.42 and 57.37 months, respectively.

### Analysis of the survival stratified by patients and tumours characteristics

Survival was first analysed regarding the following patient and tumour features: age, gender, location of the tumour, TNM stage and tumour differentiation (data on Additional file 1).

To determine whether age affects survival a cut-off point of 75 years was established. Patients older than 75 years exhibited worse prognosis, mainly in relation to OS that was 51.32 months, with a death percentage of 26.32%, while these data were of 59.32 months and 11.54%, respectively, for patients ≤75 years. This difference in OS did not reach statistical significance, although it was near to be significant (*P* = 0.082).

Gender distribution was not responsible for statistical differences in prognosis, even though women presented slightly superior recurrence and death rates.

Regarding the location of the primary tumour, both DFS and OS were higher for colon cancer patients. However, only divergence in DFS was near to the significance (*P* = 0.063), with mean values of 56.14 months and associated tumour recurrence rates of 17.39% versus 36%.

To analyse influence of TNM classification on survival, we studied DFS and OS at every TNM stage. The application of Log-Rank test rendered highly significant differences for DFS (*P* = 0.025) as well as for OS (*P* = 0.043). A TNM stage progressively more advanced correlates with a worse prognosis, both for the DFS and the OS. So, at TNM stage I recurrence was observed for just one patient and no deaths were reported, while stage II patients show percentages of tumour recurrence and death of 17.5% and 12.5% respectively, with mean DFS and OS of 55.73 and 58.55 months. Lastly, percentages of tumour recurrence and death at stage III increase to 40.9% and 27.3% respectively, with an associated DFS of 39.05 months and OS of 47.13 months.

Influence of tumour differentiation on survival was also evaluated. Survival was progressively worse from well to moderately and above all in poorly differentiated adenocarcinoma, resulting an OS statistically inferior (*P* = 0.009); recurrence was observed in 50% of patients bearing poorly differentiated tumours, while it was of 13.1% in moderately differentiated tumours and 0% in well differentiated tumours.

### Analysis of the survival of patients stratified by tumour markers

DFS and OS were analysed in patients stratified according to preoperative serum CA 72.4 and CEA levels, employing the most widely used cut-off points in clinical routine. Significant differences arised both for CA 72.4 and CEA regarding DFS as well as OS.

Table 1 reports mean DFS and OS periods, and percentages of tumour recurrence and death for the two markers in each group under analysis. Patients with preoperative serum CA 72.4 levels above the threshold of 7 U/mL exhibit recurrence and death percentages of 72.73 and 63.64%, with a mean DFS and OS of 24.11 and 31.17 months, respectively. Notably, death rate was only 6.67% for CA 72.4 levels below the cut-off. Recurrence and death rates were of 50.0% and 35.71% in patients with preoperative CEA levels over 10 ng/mL, associated with longer survival times than in the case of CA 72.4.

**Table 1:** DFS and OS rates stratified by tumour markers and univariate Cox analysis in all patients

			**D.F.S.**		**O.S.**		**Univariate Cox analysis**			
**Tumour marker**	**Levels**	**n**	**Mean (Months)**	**Recurrence%**	**Mean (Months)**	**Death%**	**Recurrence** **RH 95% CI**		**Death** **RH 95% CI**	
CA 72.4	≤ 7	60	57.12	15.00	61.23	6.67	1.00	(ref)	1.00	(ref)
U/mL	> 7	11	24.11^a^	72.73	31.17^a^	63.64	7.98	(3.03-21.00)^a^	13.37	(3.89-45.92)^a^
CEA	≤ 10	57	56.01	17.54	59.73	10.53	1.00	(ref)	1.00	(ref)
ng/mL	> 10	14	31.03^b^	50.00	37.57^c^	35.71	3.78	(1.43-10.01)^d^	4.12	(1.26-13.57)^e^

*Abbreviations:* DFS, disease free survival; OS, overall survival; RH, relative hazard; 95% CI, 95% confidence interval; ref, reference.

^a^*P* <0.0001, ^b^*P* = 0.004, ^c^*P* = 0.01, ^d^*P* = 0.007, ^e^*P* = 0.02.

To establish the recurrence and death relative hazards a univariate Cox analysis was performed. Results show that increased CA 72.4 levels imply a recurrence relative hazard of 7.98 and death relative hazard of 13.37, double and three times higher than hazards for increased CEA levels.

### Analysis of the survival of patients stratified by the positivity of tumour markers

Survival was also assessed for all patients taking into account the number of altered tumour markers, demonstrating striking differences for DFS as well as for OS when one or the two markers presented increased levels (Table 2).

**Table 2:** DFS and OS rates stratified by positivity of tumour markers and univariate Cox analysis

		**D.F.S.**		**O.S.**		**Univariate Cox analysis**			
**Positive markers**	**n**	**Mean (Months)**	**Recurrence%**	**Mean (Months)**	**Death%**	**Recurrence** **RH 95% CI**		**Death** **RH 95% CI**	
None	51	59.02	11.8	62.49	3.9	1.00	(ref)	1.00	(ref)
1	15	31.67	46.7	37.52	40.0	5.67	(1.88-17.13)^b^	12.34	(2.48-61.35)^b^
2	5	23.65 ^a^	80.0	30.23 ^a^	60.0	11.59	(3.24-41.48)^c^	23.86	(3.97-143.58)^d^

*Abbreviations:* DFS, disease free survival; OS, overall survival; RH, relative hazard; 95% CI, 95% confidence interval; ref, reference.

^a^*P* <0.0001, ^b^*P* = 0.002, ^c^*P* = 0.0002, ^d^*P* = 0.0005.

We observed that patients with normal levels of both CA 72.4 and CEA show low tumour recurrence and death rates of 11.8% and 3.9%, with a mean DFS and OS of 59.02 and 62.49 months, respectively. When one of the tumour markers exhibits increased levels the percentages of tumour recurrence and death grow to 46.7% and 40.0% (DFS and OS of 31.67 and 37.52 months), and they are dramatically increased to 80.0% and 60.0% when both markers have their levels elevated, accompanied by a reduced mean DFS of 23.65 months and OS of 30.23 months.

Univariate Cox analysis also exposed in Table 2 estimates a relative recurrence hazard of 11.59 and death hazard of 23.86 for patients with the two markers altered in comparison with patients having levels below the cut-off points.

### Multivariate analysis of the survival

Multivariate Cox analyses allow the assessment of which variables give independent prognostic information among the variables studied in relation to survival: age, gender, tumour location, TNM classification, tumour differentiation and the positivity of markers CA 72.4 and CEA (see Table 3). We obtained a model where age, tumour location, TNM stage and positivity of markers were the significant covariables regarding recurrence hazard. When looking at death hazards, only age and positivity of tumour markers remained as independent prognostic factors.

**Table 3:** Multivariate Cox analysis and relative hazards for recurrence and death in all patients

**Feature**	**Group**	**Recurrence**			**Death**		
		**RH**	**95% CI**	**P**	**RH**	**95% CI**	**P**
Age	≤75	1.00	Ref	0.021	1	Ref	0.001
	>75	3.99	(1.23-12.95)		22.19	(3.46-142.49)	
Location	colon	1.00	Ref	0.020	1.00	Ref	0.151
	rectum	3.73	(1.23-11.30)		2.65	(0.70-10.02)	
TNM	I + II	1.00	Ref	0.008	1.00	Ref	0.061
	III	4.21	(1.45-12.23)		3.66	(0.94-14.22)	
Positive markers	None	1.00	Ref		1.00	Ref	
	1	7.93	(2.34-26.87)	0.001	55.18	(6.67-456.03)	<0.001
	2	14.62	(3.78-56.58)	0.000	79.84	(8.09-788.25)	<0.001

### Analysis of the survival of patients at stage II stratified by tumour markers

**Figure 1:**
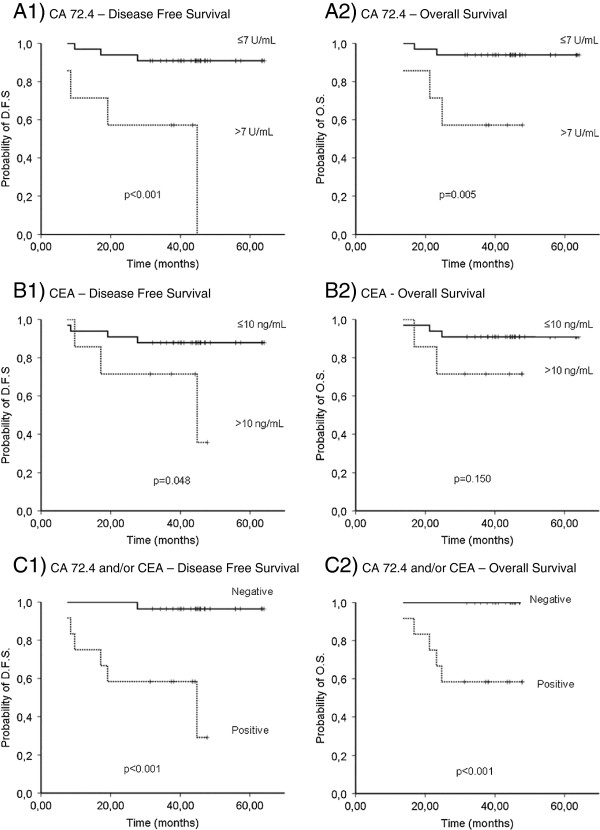
**Kaplan-Meier curves of CA 72.4 and CEA at stage II CRC.** Kaplan Meier curves of 1) the disease free survival and 2) the overall survival, for colorectal cancer patients that received curative resection of TNM stage II tumours, stratified by preoperative serum levels of **A)** CA 72.4, **B)** CEA and **C)** At least one or both markers with positive levels. D.F.S.: Disease free survival, O.S.: Overall survival. *P*: Statistical significance.

The former univariate analyses made for all patients were repeated for patients belonging to TNM stage II stratified by tumour markers. Kaplan-Meier curves for patients stratified by CA 72.4 and CEA levels are represented in Figure 1 (A1 and A2). Mean DFS, OS and the percentages of tumour recurrence and death for each marker are shown in Table 4. CA 72.4 clearly separates the two groups of patients with different DFS and OS (*P* < 0.001 and *P* = 0.005). Higher CA 72.4 levels are related to tumour recurrence and death percentages of 57.14% and 42.86%, against percentages of 9.09 and 6.06% when levels are below the cut-off. At this early stage, CEA only achieves a borderline distinction in DFS (*P* = 0.05) (Figure 1, B1 and B2).

**Table 4:** DFS and OS rates stratified by tumour markers and univariate Cox analysis at stage II

			**D.F.S.**		**O.S**		**Univariate Cox analysis**			
**Tumour marker**	**Levels**	**n**	**Mean (Months)**	**Recurrence%**	**Mean (Months)**	**Death%**	**Recurrence** **RH 95% CI**		**Death** **RH 95% CI**	
CA 72.4	≤ 7	33	59.89	9.09	61.40	6.06	1.00	(ref)	1.00	(ref)
U/mL	> 7	7	30.61^a^	57.14	35.84^b^	42.86	9.39	(2.05-43.05)^c^	8.40	(1.40-50.43)^d^
CEA	≤ 10	33	58.21	12.12	60.05	9.09	1.00	(ref)	1.00	(ref)
ng/mL	> 10	7	36.87^e^	42.86	39.89^f^	28.57	4.05	(0.90-18.23)^g^	3.44	(0.57-20.64)^h^

*Abbreviations:* DFS, disease free survival; OS, overall survival; RH, relative hazard; 95% CI, 95% confidence interval; ref, reference.

^a^*P* <0.0001, ^b^*P* = 0.005, ^c^*P* = 0.004, ^d^*P* = 0.02, ^e^*P* = 0.05, ^f^*P* = 0.15, ^g^*P* = 0.07, ^h^*P* = 0.17.

The univariate Cox analysis exposed in Table 4 summarizes the relative recurrence and death hazards for patients at stage II classified by preoperative serum CA 72.4 or CEA levels. Those patients presenting CA 72.4 levels higher than 7 U/mL have relative recurrence and death hazards of 9.39 and 8.40, whilst CEA exerts no significant influence on these hazards.

### Analysis of the survival of patients at stage II stratified by the positivity of tumour markers

Data on DFS and OS, and univariate Cox analysis comparing the group of patients with at least one marker altered versus reference group patients with no altered levels of any tumour marker are displayed in Table 5. Only one case of tumour recurrence and none of death were reported on patients without increased levels of the markers (DFS: 62.77 months). Prognosis worsens for stage II patients with overexpressed levels of CA 72.4, CEA or both, showing percentages of tumour recurrence and death of 50% and 41.67%, associated with a very low mean DFS and OS of 32.15 and 36.20 months, respectively. The relative recurrence hazard was 21.52 times higher than in patients with no altered levels. The death hazard ratio could not be calculated because there was not any death in the group without positivity for any tumour marker. Figure 1, C1 and C2 represent Kaplan-Meier survival curves of this last analysis.

**Table 5:** DFS and OS rates stratified by positivity of tumour markers and univariate Cox analysis at stage II

		**D.F.S.**		**O.S.**		**Univariate Cox analysis**			
**Positive markers**	**n**	**Mean (Months)**	**Recurrence%**	**Mean (Months)**	**Death%**	**Recurrence** **RH 95% CI**		**Death** **RH 95% CI**	
None	28	62.77	3.57	-	0	1.00	(ref)	1.00	(ref)
CEA and/or CA 72.4	12	32.15^a^	50.0	36.20 ^a^	41.67	21.52^b^	(2.53-182.86)	-	-

^a^*P* <0.001, ^b^*P* <0.01.

## Discussion

CRC clinical research aims to obtain novel tools to improve prognosis giving light in the decision-making regarding adjuvant chemotherapy. Specifically, identification of reliable factors that improve selection of stage II patients at high-risk of developing recurrence after surgery is of greatest importance [9]. In the current study, we assessed the relevance of CA 72.4 in prognosis when measured in serum of patients before curative surgery.

DFS rates obtained in this study for TNM stages I-III were highly similar to the ones obtained for Dukes staging, by others and in previous works of us, although rates of death were a bit lower provided this work has not included patients at TNM stage IV and it is a 5-year survival study [24-27].

Preoperative serum values of CA 72.4 proved its role as prognostic factor, with significantly higher recurrence and death percentages for levels above 7 U/mL. These percentages exceeded the displayed by CEA, the standard for CRC prognosis [28,29]. Univariate Cox analysis corroborated the superior value of prognostic information provided by CA 72.4, as recurrence and death are more prone to occur with altered CA 72.4 levels. Nevertheless, CEA complements the prognostic information offered by CA 72.4 since recurrence and death are better predicted when both markers are altered in the patients, being this finding in agreement with the work of Louhimo [24].

Although studies on CA 72.4 in preoperative serum in patients of CRC are very scarce [24,30], results agree on the prognostic value of this marker. However, its performance complementing CEA is not well established.

Multivariate Cox analysis was used to generate a model that best explains the recurrence and death relative hazards and test the prognostic independence of the variables that influenced survival in univariate analysis. In addition to TNM, age, tumour location and positivity of tumour markers resulted significant. Positivity of tumour markers is the strongest predictor for recurrence and death.

The current study aimed also to assess the prognostic relevance of CA 72.4 at TNM stage II, to discriminate those patients who are definitely cured after having a curative resection of the tumour, from those that will relapse or even die. Survival analysis confirmed the precise distinction of patients with different DFS and OS by CA 72.4, whilst CEA has a non-significant effect on the recurrence and death hazards, at this early stage. Nevertheless, CEA contribution to prognosis prediction is not negligible, as shows the analysis taking into account the number of positive markers.

For years, efforts have been made on the search for more effective predictors than the traditional staging system not only across all CRC stages, but also regarding early-stage CRC. Genetic and molecular markers that could be useful for detecting who is or not at risk emerged earlier [3,4,31-36]. Nowadays, a different approach based on the identification of clustered genetic alterations and multimarker phenotypes is gaining wider acceptance [36-38].

Conversely, studies dividing the TNM-stages into subgroups are rare. For instance, in a recent review to study the clinical significance of circulating tumour cells in non-metastatic CRC, Thorsteinsson and Jess [39] revised 9 studies and none of them had divided the TNM-stages into subgroups. Recently, a multigene expression assay has been developed to determine the relationship between quantitative tumour gene expression and the risk of cancer recurrence at stage II. This quantitative multigene expression assay is now marketed as Oncotype DX Colon Cancer assay [40].

Unfortunately, to date, all these advances are not yet being translated to the clinical routine, giving arguments for implementation of CA 72.4 into clinical practice. The present research has proved its prognostic relevance that could complement the actual clinical classification of patient prognosis based on the TNM system. This marker has also proved to be valid to determine which patients at stage II are in the high-risk category and therefore should be given chemotherapy, and those who will not have recurrent disease and are therefore in the low-risk category. Moreover, in comparison with genetic markers, determination of CA 72.4 preoperative levels is simple, without necessity of available tissue and cost-effective, all of this corroborated by the fact that is already employed in gastric cancer routine prognosis.

## Conclusions

The present work has proved the prognostic relevance of CA 72.4 at stage II CCR, and how in combination with CEA allows the correct classification of nearly every patient with bad prognosis. This could improve the decision-making involved in the treatment of stage II colon cancer.

Further studies to assess the possible role of the marker in the prediction of the effectiveness of treatment are worthy.

## Abbreviations

CA 72.4: Carbohydrate antigen 72,4; CEA: Carcinoembryonic antigen; CRC: Colorectal cancer; DFS: Disease free survival; OS: Overall survival; TNM: Tumour, Node and Metastasis.

## Competing interests

The authors declare that they have no competing interests.

## Authors’ contributions

Experiments were conceived and designed by FJRB, DA and MPDC. DA, JA and MVC participated in the acquisition of data. MPDC, DA, SBP, JA, MVC and LVI made significant contributions to the acquisition of data, critical revision of the manuscript and drafting of the manuscript. FJRB, MPDC and DA made critical revision of the manuscript for important intellectual content. DA, JA and MVC participated in the statistical analysis. All authors read and approved the final manuscript.

## Pre-publication history

The pre-publication history for this paper can be accessed here:

http://www.biomedcentral.com/1471-2407/13/543/prepub

## Supplementary Material

Additional file 1: Table S1 Univariate survival analysis. Analysis of the survival stratified by patients and tumours characteristics. Click here for file
