# Supplementary material for: Preoperative serum CA 72.4 as prognostic factor of recurrence and death, especially at TNM stage II, for colorectal cancer
Source: BMC Cancer. 2013 Nov 12;13:543. doi: 10.1186/1471-2407-13-543 (PMC3829802; doi:10.1186/1471-2407-13-543)
Supplement: Additional file 1: Table S1 — Univariate survival analysis. Analysis of the survival stratified by patients and tumours characteristics. [file 1471-2407-13-543-S1.docx]

**Additional file 1**

**Table S1- Univariate survival analysis:** Analysis of the survival stratified by patients and tumours characteristics

|  |  | | **DFS** | | | | **OS** | |
| --- | --- | --- | --- | --- | --- | --- | --- | --- |
|  | | N | | **Mean**  **(Months)** | **%**  **Recurrence** | **Mean**  **(Months)** | | **%**  **Death** |
| **Age** | |  | |  |  |  | |  |
| ≤75 | | 52 | | 54.8 | 21.15 | 59.32 | | 11.54 |
| >75 | | 19 | | 46.87 | 31.58^a^ | 51.32 | | 26.32^b^ |
| **Gender** | |  | |  |  |  | |  |
| male | | 38 | | 54.04 | 21.05 | 58.00 | | 13.16 |
| female | | 33 | | 50.33 | 27.27^c^ | 56.01 | | 18.18^d^ |
| **Location** | |  | |  |  |  | |  |
| colon | | 46 | | 56.14 | 17.39 | 58.75 | | 10.87 |
| rectum | | 25 | | 40.81 | 36.00^e^ | 49.37 | | 24.00^f^ |
| **TNM** | |  | |  |  |  | |  |
| I | | 9 | | 50.83 | 11.11 | -* | | 0 |
| II | | 40 | | 55.73 | 17.5 | 58.55 | | 12.5 |
| III | | 22 | | 39.05 | 40.9^g^ | 47.13 | | 27.3^h^ |
| **Tumor Differentiation** | |  | |  |  |  | |  |
| well | | 4 | | - | 0 | - | | 0 |
| moderately | | 61 | | - | 23 | - | | 13.1 |
| poorly | | 6 | | - | 50^i^ | - | | 50.0^j^ |

Abbreviations: DFS, disease free survival; OS, overall survival.

*Could not be estimated

^a^ *P* = 0.242, ^b^ *P*=0.082, , ^c^ *P* =0.566 0,^d^*P* =0.554 , ^e^ *P* = 0.063, ^f^ *P*=0.194, , ^g^ *P* =0.025,^h^*P* =0.043, ^i^ *P* =0.129,^j^*P* =0.009
